# “Vegan” and “plant-based” claims: risk implications for milk- and egg-allergic consumers in Canada

**DOI:** 10.1186/s13223-023-00836-w

**Published:** 2023-08-24

**Authors:** Silvia Dominguez, Jérémie Théolier, Kamila Lizée, Beatrice Povolo, Jennifer Gerdts, Samuel B. Godefroy

**Affiliations:** 1 Food Risk Analysis and Regulatory Excellence Platform (PARERA), Institute of Nutrition and Functional Foods and Department of Food Science, Université Laval, 2440 Hochelaga Boulevard, Quebec, QC G1V 0A6 Canada; 2 Food Allergy Canada, 505 Consumers Drive, Suite 507, Toronto, ON M2J 4A2 Canada

**Keywords:** Vegan, Plant-based, Food labelling, Egg allergy, Milk allergy, Survey, Risk

## Abstract

The market share of foods carrying labels suggesting absence of animal ingredients has significantly increased in recent years. The purpose of this study was to document the purchasing behaviour of egg- or milk-allergic consumers vis-à-vis food marketed as “vegan” and “plant-based”, and to evaluate the associated allergenic risk. A survey was conducted among egg- and milk-allergic consumers and revealed that 86% (margin of error 5.4%; confidence level 95%) would buy “vegan” products, indicating this label may be perceived as a substitute for “free from milk and eggs”. To assess the risk posed by these products, 87 prepackaged foods with “vegan” and/or “plant-based” claims purchased in Quebec were tested for milk proteins, and 64 for egg proteins. Overall, 5.7% and 0% occurrence of milk and egg proteins, respectively, were observed, suggesting that the analysed prepackaged foods carrying “vegan” and/or “plant-based” labels pose little risk to egg- or milk-allergic consumers. However, this is likely due to allergen management practices applied by the Canadian manufacturers of the products tested, and should not be attributed to the use of “vegan” or similar labels. Enhanced regulatory requirements for the use of these labels, and an education campaign on their meaning with respect to allergy-related risks, are necessary to better inform and protect egg- and milk-allergic consumers.

**Supplementary Information:**

The online version contains supplementary material available at 10.1186/s13223-023-00836-w.

## Introduction

The availability of prepackaged foods marketed as “vegan” has significantly increased in the last decades, reaching a global market value of more than USD 24 billion in 2022 [1]. This growth is expected to continue, driven by consumers’ increasing concerns related to the environment, sustainability, and animal welfare, as well as their interest in foods perceived as healthier [2]. When referring to foods, the term “vegan” generally implies that ingredients of animal origin (meat, poultry, fish, seafood), including eggs and milk, are not part of the formulation [3]. Individuals allergic to animal proteins (e.g., milk, egg) may therefore perceive “vegan” labels as indicators of safety and use them to guide purchasing decisions.

Although “vegan” certifications and standards (ISO 23662:2021) for prepackaged foods exist, they do not guarantee the absence of cross-contact allergens of animal origin. More importantly, this term is generally not specifically defined in food regulations. In Canada, the Canadian Food Inspection Agency (CFIA) allows for some flexibility in its interpretation and notes that “While a vegan diet or foods are made from only plant-based ingredients, it is also recognized that several definitions of ‘vegan’ exist. When making claims on a food, companies can apply additional criteria or standards that take account of other factors in addition to the ingredients of the food” [4]. Subsection 5 (1) of the Food and Drugs Act [5] requires information on food labels to be “truthful and not misleading” and therefore applies to products marketed as “vegan”, but its enforcement in this context is not feasible in the absence of a regulatory definition or standard. Yet, most CFIA recalls of products marketed as “vegan” are due to presence of undeclared ingredients of animal origin (19/21, since 2012), notably milk and egg [6]. Similarly, cases of “vegan” products containing these allergens have been reported in several jurisdictions other than Canada [3, 7, 8], posing a serious risk to allergic consumers, and in one instance, a fatality [9].

In addition, “plant-based” and related food labels are experiencing explosive growth worldwide, with a forecasted market share around USD 34 billion in 2024 [10]. These foods are expected to be made primarily of plant-based ingredients, and are gaining popularity as a viable alternative to animal proteins in terms of cost, nutrition and sustainability. However, foods marketed as “plant-based” may be formulated to comply with diets other than “vegan”, such as “vegetarian” (similar to “vegan” but may include eggs, dairy products or honey), “ovo-lacto-vegetarian” (vegetarian with the inclusion of eggs and dairy) or even “flexitarian” (vegetarian with occasional inclusion of animal products), and thus do not by definition exclude animal ingredients [10]. Some plant-based proteins have been identified as potential novel allergens (e.g., pulses) [11, 12]; yet “plant-based” foods may appeal to consumers allergic to animal proteins [13]. Requirements for specific “plant-based” foods and beverages are considered in Canadian regulations, mainly addressing nutritional value and product naming [14, 15], but provisions related to animal ingredients content are not included.

Thus, the objectives of this study were (i) to report on the buying behaviour of egg- and milk-allergic consumers in Canada related to products containing a “vegan” claim, and (ii) to draw a preliminary overview of the occurrence of egg and/or milk proteins in this food category. Fish and shellfish allergens were not considered in this study because they have not been the cause of food recalls to date, and because “vegan” products carrying precautionary allergen labelling (PAL) for them were not found in the market.

## Materials and methods

### Consumer survey

An online survey of Canadian adults with (or parents of a child with) a food allergy was conducted in 2021 as described in Graham et al. (2023) [16]. A specific question was included in this survey (but not reported in Graham et al. [16]), only for respondents that indicated having an allergy to (or being a parent of a child with an allergy to) egg or milk: *When buying food products you will consume (or that will be consumed by your child with and allergy to egg or milk), how often, if ever, do you purchase products containing a vegan claim?* Answer choices were: *Never; Sometimes/Depends on situation; Always*. Respondents could select one answer only.

### Market survey

#### Sample collection

Food samples were randomly purchased from three different supermarkets in Quebec, Canada between March and December 2022. Items that contained the labels “vegan” or “plant-based” were targeted, regardless of their certification status. For instance, products with “vegan” on the name and products with a “certified vegan” logo somewhere on the package were classified simply as “vegan” in this study. If a “vegan” product also contained a “plant-based” claim, it was classified only as “vegan”. In some cases, the number of items sampled from the same brand was intentionally restricted, given that similar items with little modifications were found (e.g., snack bars from the same brand clearly belonging to the same product line, only with flavor variations). In this case, items with PAL for milk and/or egg, items with a longer list of ingredients, or items containing chocolate ingredients, were selected. In addition, overall, products carrying the targeted claims and also PAL for milk and/or egg were purposely selected. All types of PAL statements were considered, including but not limited to “may contain [allergen]”, “made in a facility that processes [allergen]”, “may have been in contact with [allergen]”, as well as statements including egg and/or milk along with several other allergens. In each of the retail points visited, items from all food categories (Additional file 1: Tables S1 and S2) that included the targeted labels were sampled. All samples were transported to the laboratory immediately after purchasing, stored in their original packaging, according to the instructions on the label, and analysed before their expiration dates.

#### Sample preparation

If products contained individually packed components (e.g., sauce and pasta), each component was analysed separately. For most samples, about half of the contents of each food product was ground using a Grindomix GM200 (Retsch, Haan, Germany). For products or their subcomponents packaged in units containing less than 20 g (e.g., sauce), the entire package content was ground. Frozen products were stored at 4 °C overnight before grinding.

#### ELISA methods

Food samples were analysed in duplicates using the RIDASCREEN®FAST Milk and RIDASCREEN®FAST Egg from R-Biopharm (R-Biopharm AG, Darmstadt, Germany). Results were analysed using the RIDASOFT^®^Win.NET software and considered positive when above the limit of quantification, corresponding to 2.5 mg/kg for milk proteins and 0.245 mg/kg for egg proteins. Samples containing components potentially subject to little or no heat treatment (e.g., low acid spreads and sauces, cookie dough, fresh pasta, not-ready-to-eat multi component foods) as well some fully cooked items (e.g., cookies, crackers, ready-to-eat meals) were tested for egg. The latter were included with the intention of detecting post-processing cross-contact egg, if present, since the kit used is recommended for the detection of raw egg proteins [17, 18]. Manufacturer instructions for sample preparation and analysis were followed for both kits. Depending on the type of food product, samples were tested for both allergens (e.g., imitation meat), for milk only (e.g., samples containing chocolate) or for egg only (e.g., imitation egg).

## Results

### Consumer survey

Of the total sample of 1080 questionnaires completed, 20% (211) indicated having an allergy to (or being a parent of a child with an allergy to) egg and 20% (211) to milk (further details in Graham et al. [16]). The question of interest to this study (*When buying food products you will consume – or that will be consumed by your child with and allergy to egg or milk –, how often, if ever, do you purchase products containing a vegan claim?)* was answered by 337 respondents as follows: *Never* (14%); *Sometimes / Depends on situation* (72%)*; Always* (14%). Based on Clarke et al. (2019), the estimated prevalence for egg allergy is 0.8% in the Canadian population, and 1.1% for milk [19]. The estimated Canadian population is about 37 million [20]. Consequently, the sample size of survey responses provides a margin of error of 5.4% at a 95% confidence level.

### Market survey

A total of 124 distinct prepackaged food samples, from 87 different brands (86% from Canada / United States, 8% from Europe and 6% from Asia), were included in this study. Most samples belonged to the “vegan” group (111/124). Among these, 18% had PAL for milk and 12% for egg (Table 1). A limited number of products marketed as “plant-based”– but not “vegan”—were found, with an 8% prevalence of PAL for milk and egg among these samples (Table 1).

**Table 1:** Samples included in this study and prevalence of PAL^a^ for milk and/or egg

Claim^b^	No. of samples	No. of samples with PAL for milk	No. of samples with PAL for egg
“Vegan”	111	20	13
“Plant-based” (but not “vegan”)	13	1	1
Total	124	21	14

^a^All types of precautionary statements were considered (e.g., “may contain [allergen]”, “made in a facility that processes [allergen]”). The same product may carry PAL for milk and egg

^b^On the product’s name and/or in a logo, with or without certification

Of the 124 samples collected, 87 were tested for milk proteins and 64 for egg proteins (Additional file 1: Tables S1 and S2). Egg proteins were not detected in 100% of 64 samples analysed. Milk proteins were detected in 5/87 samples analysed: 4 distinct “certified vegan” dark chocolate bars from the same manufacturer (134.9 ± 18.5 ppm) carrying PAL for milk, and 1 “vegan” supermarket brand cake—a chestnut Christmas log (2.6 ppm), listing milk in a blanket PAL statement (i.e., “may contain or may have been in contact with soy, peanuts, nuts, mustard, sesame, yeast, wheat and triticale, eggs, milk”). Considering one entire chocolate bar (0.035 kg, per product label) is likely to be consumed in one eating occasion, the mean concentration of cross-contact milk proteins detected would represent an exposure dose of 4.72 mg. This dose could elicit a reaction in approximately 6% of milk-allergic consumers [21], and is more than twice the milk reference dose (2 mg) recommended by the FAO/WHO expert consultation on food allergens [22]. The “vegan” cake’s label indicated it contained three servings. Thus, the cake’s total weight of 0.29 kg was divided by 3 to estimate the amount that would be consumed in one eating occasion (0.097 kg). The concentration of cross-contact milk proteins detected in this sample would then represent an exposure dose of 0.25 mg. This dose could elicit a reaction in approximately 1% of milk-allergic consumers [21], and is below the reference dose recommended by the FAO/WHO expert consultation [22].

## Discussion

Consumer survey results indicate that 86% of respondents reporting an allergy to egg or milk would buy products with a “vegan” claim. This strongly suggests “vegan” labels may be used as indicators of safety by consumers allergic to animal proteins (i.e., milk, egg) and use them to guide purchasing decisions. This is the first time this buying behaviour is formally documented. In 2017, Marchisotto et al. [23] reported that about 40% of North American allergic consumers would buy products with PAL, while Graham et al. (2023) [16] observed that these numbers are on the rise in Canada, with 54% of allergic consumers reporting this purchasing behaviour. As the presence of PAL is directly correlated with the potential presence of an allergen hazard, it is not surprising that consumers would prefer to buy a product with a “vegan” claim—probably perceived as “absence of milk and eggs”—than one with PAL for these allergens. The number reported in this study (86% likelihood of buying “vegan” products) strongly suggests that respondents correlated the claim “vegan” with a low level of risk, potentially making it a credible indicator for allergic consumers. This is a matter of concern, as “vegan” claims should not prevail to good allergen management practices and should not be perceived as such by consumers or manufacturers. This indicates that allergic consumers are not fully aware of the food labelling regulatory framework, as previously reported [23–25], and that there is a need for credible indicators directly linked to the level of cross-contact allergen risk, as suggested by the FAO/WHO expert consultation on food allergens [26]. The use of this type of indicator (e.g., a logo indicating the food manufacturer conducted an allergen risk assessment) would prevent allergic consumers from drawing conclusions based on unregulated claims like “vegan”.

Although the consumer survey focused on “vegan” labels only, products with “plant-based” (but not “vegan”) labels were also included in the market survey due to their growing market presence and the closeness of both labels’ target audiences. Furthermore, the CFIA website lists 11 recalls related to “plant-based” foods associated with undeclared ingredients (i.e., milk, egg) since 2012, 8 of which were Class 1 (i.e., based on a risk assessment, the agency considered that there was a high risk that consuming the food may lead to serious health problems or death) [27].

Market survey results indicate overall 5.7% and 0% occurrence of milk and egg proteins, respectively. Four out of 5 milk-positive samples were dark chocolate bars, containing milk proteins at relatively high levels (134.9 ± 18.5 ppm; 4.72 mg estimated exposure dose). This type of cross-contact has been previously reported and discussed [7, 28–30]. Although contradictory with the general perception of a “vegan” food product, the use of PAL for milk in the chocolate bars tested in this survey is necessary to inform allergic consumers of the risk of cross-contact milk. Yet, the use of PAL along with a more visible (i.e., front label) “certified vegan” statement may mislead consumers and decrease its efficacy as a risk management tool. Milk proteins were also detected in a chestnut cake, with the text “vegan” in the product’s name (i.e., “vegan chestnut cake”) and milk in a blanket PAL statement. Although milk protein levels were lower in this sample (2.6 ppm; 0.25 mg estimated exposure dose), this finding brings up attention to the potential issue of cross-contact milk beyond dark chocolate noted in other market surveys [3, 17, 31, 32]. Yang et al. [3] included “vegan” items (n = 19) in their market survey of cross-contact milk in milk-alternative frozen desserts; all these samples were below the level of quantification, as was the case for all items of this food category (n = 5) included in our survey. In addition, although applicable to a different jurisdiction (United States), Yang et al. (2022) [3] also raised the issue of potentially contradictory labelling (e.g., “vegan” items with PAL for milk) and the risk it may pose to consumers seeking to avoid milk. No other market surveys of cross-contact allergens in “vegan” products were found in the literature. Although not as often as milk, undeclared egg has also been previously reported [17, 18, 31], but mostly in cooked items (e.g., baked goods, snacks, fishery items), with no mention of “vegan” labels. Thus, our results are not comparable. Future studies could investigate the occurrence of cross-contact egg in thermally treated “vegan” or “plant-based” items, and thus expand our survey, which targeted detection of egg in food products subject to little or no heat treatment. This however would require the selection of a fit for purpose analytical method, given that the test kit used in our study (RIDASCREEN®FAST Egg) is not indicated for this purpose and may therefore result in underestimation [18].

Overall, the results of this market survey indicate that, in Quebec, prepackaged foods carrying “vegan” or “plant-based” labels pose little risk to egg- or milk-allergic consumers. However, given the high prevalence of Canadian brands among the products surveyed (51/84), this is most likely due to allergen management practices applied by this industry [33], and should not be attributed to the use of “vegan” or similar labels. These claims are not regulated with respect to the content of ingredients of animal origin and must not be interpreted as such by consumers allergic to animal proteins. It is possible that the high prevalence (more than 80%) of egg- and milk-allergic individuals choosing to consume “vegan” foods is due to a lack of awareness of regulatory requirements associated with this claim coupled with absence or very low levels of cross-contact egg and milk proteins in most of these items, reinforcing their misperception of guaranteed safety. This behaviour may be compared to previous reports [28, 34] of allergic individuals consuming products with PAL because they did not experience an allergic reaction after testing a small portion, and incorrectly assuming these products will consistently deliver the same level of safety. Thus, a consumer education campaign on the regulatory meaning of “vegan” and similar labels, highlighting the potential presence of cross-contact allergens, and therefore PAL, may be warranted, especially considering that not all food manufacturers adhere to allergen management practices equivalent to those applied in Canada. On the other hand, manufacturers should be aware of how some allergic consumers interpret “vegan” claims (e.g., absence of ingredients of animal origin) and avoid specifically targeting this population (e.g., avoid using statements like “allergy friendly”—as noted in one item in this survey). The working definition of “vegan” provided by CFIA indicates that criteria other than the food’s ingredients may be considered when using this claim [4]. Consequently, the presence of ingredients of animal origin or the use of PAL for these is beyond the scope of regulatory enforcement. Adherence to a “vegan” diet usually reflects a consumer’s ideological concerns and is not normally intended to prevent acute health issues such as food allergies. However, the fact that some allergic consumers may use “vegan” claims as indicators of a product’s safety should concern regulatory authorities. For example, in some cases, this claim may be seen as an alternative to “dairy-free” by manufacturers and/or consumers, but the risk to milk-allergic consumers is not comparable.

Limitations of this market survey include a potential underestimation of egg proteins in baked items, considering that the kit used is better suited for the detection of raw egg proteins. In addition, the market survey’s scope is geographically limited (only foods sold in Quebec, Canada). Nevertheless, it provides an overview of the occurrence of two of the main allergens of animal origin (i.e., egg and milk) in most food groups carrying “vegan” or “plant-based” labels. These results however must not be extrapolated to foods sold in bulk with similar claims, which may present a different level of risk [35].

## Conclusions

Egg- or milk-allergic consumers include “vegan” products in their buying choices, suggesting they perceive them as safe. The products analysed in this study posed little or no risk to consumers allergic to egg or milk proteins. This however should not be attributed to “vegan” or “plant-based” claims, which are not regulated in Canada to indicate the absence of the relevant offending allergen and are not intended to communicate risks to consumers. Instead, our analytical results may be reflective of allergen management practices applied by Canadian food manufacturers in general. Efforts should be considered to enhance specific regulatory requirements for the use of “vegan” and similar claims, including provisions related to the implementation of appropriate allergen control plans. Finally, an education campaign on the meaning of “vegan” and similar claims with respect to allergenic risk is necessary to better inform this population’s food purchasing strategies.

### Supplementary Information

**Additional file 1:** Description of the “vegan” and “plant-based” food products included in the market survey, analysed for milk proteins (**Table S1**) and egg proteins (**Table S2**).

## Data Availability

All data generated or analysed during this study are included in this published article [and its supplementary information files].

## Abbreviations

PAL: Precautionary allergen labelling
CFIA: Canadian Food Inspection Agency
FAO: Food and Agriculture Organization of the United Nations
WHO: World Health Organization
