# Supplementary material for: “Vegan” and “plant-based” claims: risk implications for milk- and egg-allergic consumers in Canada
Source: Allergy Asthma Clin Immunol. 2023 Aug 24;19:74. doi: 10.1186/s13223-023-00836-w (PMC10464346; doi:10.1186/s13223-023-00836-w)
Supplement: Supplementary file 1 — Additional file 1: Description of the “vegan” and “plant-based” food products included in the market survey, analysed for milk proteins (Table S1) and egg proteins (Table S2). [file 13223_2023_836_MOESM1_ESM.docx]

Supplementary materials

Supplementary Table 1. Summary of products sampled and analysed for milk proteins

| **Food category** | **Number of samples** | **Number of brands** | **Number of products with precautionary statement^(1)^ for:** | |
| --- | --- | --- | --- | --- |
|  |  |  | **Milk** | **Egg** |
| **Products marketed as “vegan”^(2)^** | | | | |
| Baked goods | 9 | 9 | 1 | 2 |
| Baking mix | 1 | 1 | 0 | 0 |
| Beverage | 1 | 1 | 0 | 0 |
| Cereals and grains | 1 | 1 | 0 | 0 |
| Cookies, crackers, and snacks | 8 | 5 | 0 | 0 |
| Dairy alternatives |  |  |  |  |
| Butter | 1 | 1 | 0 | 0 |
| Cheese | 7 | 6 | 0 | 0 |
| Cream | 3 | 2 | 0 | 0 |
| Frozen dessert | 4 | 2 | 0 | 0 |
| Milk | 1 | 1 | 0 | 0 |
| Yogurt | 4 | 3 | 1 | 0 |
| Dark chocolate | 4 | 1 | 4 | 0 |
| Egg alternatives | 1 | 1 | 0 | 1 |
| Frozen meals | 6 | 5 | 0 | 0 |
| Meat alternatives | 7 | 7 | 0 | 0 |
| Pasta | 4 | 2 | 1 | 1 |
| Ready-to-Eat meals | 2 | 1 | 2 | 2 |
| Sauce | 5 | 5 | 1 | 1 |
| Soups | 3 | 2 | 2 | 0 |
| Other | 5 | 5 | 0 | 0 |
| **Sub-TOTAL** | **77** | **61** | **12** | **7** |
|  |  |  |  |  |
| **Products marketed as “plant-based”^(2)^ (but not “vegan”)** | | | | |
| Cookies, crackers, and snacks | 2 | 2 | 1 | 0 |
| Dairy alternatives |  |  |  |  |
| Cheese | 1 | 1 | 0 | 0 |
| Frozen dessert | 1 | 1 | 0 | 0 |
| Egg alternatives | 2 | 2 | 0 | 1 |
| Fish alternatives | 1 | 1 | 0 | 0 |
| Meat alternatives | 3 | 2 | 0 | 0 |
| **Sub-TOTAL** | **10** | **9** | **1** | **1** |
|  |  |  |  |  |
| **TOTAL** | **87** | **70** | **13** | **8** |

1. All types of precautionary statements were considered (e.g., “may contain [allergen]”, “made in a facility that processes [allergen]”). The same product may carry PAL for milk and egg.
2. On the product’s name or in a logo, with or without certification.

Supplementary Table 2. Summary of products sampled and analysed for egg proteins

| **Food category** | **Number of samples** | **Number of brands** | **Number of products with precautionary statement^(1)^ for:** | |
| --- | --- | --- | --- | --- |
|  |  |  | **Milk** | **Egg** |
| **Products marketed as “vegan”^(2)^** | | | | |
| Baked goods | 5 | 4 | 2 | 3 |
| Beverage | 2 | 2 | 0 | 0 |
| Candy | 1 | 1 | 0 | 0 |
| Cereals and grains | 2 | 2 | 0 | 0 |
| Cookies, crackers, and snacks | 17 | 16 | 4 | 5 |
| Dairy alternatives |  |  |  |  |
| Butter | 1 | 1 | 0 | 0 |
| Cheese | 6 | 5 | 0 | 0 |
| Cream | 2 | 2 | 0 | 0 |
| Frozen dessert | 2 | 2 | 0 | 0 |
| Yogurt | 1 | 1 | 0 | 0 |
| Dark chocolate | 1 | 1 | 1 | 0 |
| Frozen meals | 2 | 2 | 0 | 0 |
| Meat alternatives | 3 | 3 | 0 | 0 |
| Pasta | 2 | 2 | 1 | 1 |
| Ready-to-Eat meals | 2 | 1 | 2 | 2 |
| Sauce | 3 | 3 | 1 | 1 |
| Salad dressing | 2 | 2 | 0 | 0 |
| Other | 2 | 2 | 1 | 0 |
| **Sub-TOTAL** | **56** | **52** | **12** | **12** |
|  |  |  |  |  |
| **Products marketed as “plant-based”^(2)^ (but not “vegan”)** | | | | |
| Baked goods | 1 | 1 | 0 | 0 |
| Cookies, crackers, and snacks | 2 | 2 | 1 | 0 |
| Dairy alternatives |  |  |  |  |
| Frozen dessert | 1 | 1 | 0 | 0 |
| Egg alternatives | 2 | 2 | 0 | 1 |
| Fish alternatives | 1 | 1 | 0 | 0 |
| Pasta | 1 | 1 | 0 | 0 |
| **Sub-TOTAL** | **8** | **8** | **1** | **1** |
|  |  |  |  |  |
| **TOTAL** | **64** | **60** | **13** | **13** |

1. All types of precautionary statements were considered (e.g., “may contain [allergen]”, “made in a facility that processes [allergen]”). The same product may carry PAL for milk and egg. Products carrying PAL for milk and/or egg along with other allergens were also included.
2. On the product’s name and/or in a logo, with or without certification.
